# Diabetic foot: prevalence, knowledge, and foot self-care practices among diabetic patients in Dar es Salaam, Tanzania – a cross-sectional study

**DOI:** 10.1186/s13047-015-0080-y

**Published:** 2015-06-05

**Authors:** Faraja S. Chiwanga, Marina A. Njelekela

**Affiliations:** Muhimbili National Hospital, Kalenga Street, Upanga, P.O Box 65000, Dar es Salaam, Tanzania; Muhimbili University of Health and Allied Sciences, Mindu Street, Upanga, P.O Box 65001, Dar es Salaam, Tanzania

**Keywords:** Diabetes, Diabetic foot, Foot care

## Abstract

**Background:**

At the time of diagnosis, more than 10 % of people with type 2 diabetes mellitus have one or two risk factors for a foot ulceration and a lifetime risk of 15 %. Diabetic foot ulcers can be prevented through well-coordinated foot care services. The objective of this study was to determine knowledge of foot care and reported practice of foot self-care among diabetic patients with the aim of identifying and addressing barriers to preventing amputations among diabetic patients.

**Methods:**

Patients were randomly selected from all public diabetic clinics in Dar es Salaam. A questionnaire containing knowledge and foot care practice questions was administered to all study participants. A detailed foot examination was performed on all patients, with the results categorized according to the International Diabetes Federation foot risk categories. Statistics were performed using SPSS version 14.

**Results:**

Of 404 patients included in this study, 15 % had foot ulcers, 44 % had peripheral neuropathy, and 15 % had peripheral vascular disease. In multivariate analysis, peripheral neuropathy and insulin treatment were significantly associated with presence of foot ulcer. The mean knowledge score was 11.2 ± 6.4 out of a total possible score of 23. Low mean scores were associated with lack of formal education (8.3 ± 6.1), diabetes duration of < 5 years (10.2 ± 6.7) and not receiving advice on foot care (8.0 ± 6.1). Among the 404 patients, 48 % had received advice on foot care, and 27.5 % had their feet examined by a doctor at least once since their initial diagnosis. Foot self-care was significantly higher in patients who had received advice on foot care and in those whose feet had been examined by a doctor at least once.

**Conclusions:**

The prevalence of diabetic foot is high among patients attending public clinics in Dar es Salaam. There is an urgent need to establish coordinated foot care services within the diabetic clinic to identify feet at risk, institute early management, and provide continuous foot care education to patients and health care providers.

## Background

It is estimated that, at the time of diagnosis of type 2 diabetes mellitus, more than 10 % of patients have one or two risk factors for foot disease, such as peripheral neuropathy (PN) or peripheral vascular disease (PVD) [1]. The burden of diabetic foot disease is expected to increase given the increasing global prevalence of T2DM. Worldwide, 3 %–10 % of people with diabetes have a foot ulcer (DFU); the lifetime risk for developing DFU is 15 % [1]. Rates of foot ulceration in Africa vary between regions and have been estimated to be between 4 % and 19 % [2].

Several studies have shown that a majority of people with diabetes do not receive guideline-recommended foot care, including regular foot examinations. [3] In a study conducted by Basu et al. in the United Kingdom, 33 % of people with diabetes did not recall receiving information about foot care [4]. In a study conducted at the Muhimbili National Hospital (MNH) diabetic clinic, 87 % of patients reported never inspecting their feet, and 66 % reported they were not interested in further knowledge of diabetes foot care [5].

Among the complications of diabetes, lower limb amputation is considered to be potentially preventable. [6] Most lower limb amputations in patients with diabetes are preceded by a foot ulcer, whose risk factors apart from PVD and PN, are barefoot walking, inappropriate footwear, poor foot hygiene and delay in seeking medical attention [7]. These non-traditional risk factors can be modified if identified early, and if patients have adequate knowledge of foot care and put that knowledge into practice. [7] In recent years, the level of interest and knowledge about DFU has grown considerably, as witnessed by the development of an international consensus, clinical guidelines to be used in both prevention and treatment of diabetic foot, and improvements in evidence-based clinical practice. All patients, if given proper guidance and education regarding foot care, should be able to make significant improvements in their foot care. Evidence that foot care education alone prevents DFU and amputation has been inconsistency due to lack of high quality randomized trials. However, this lack of evidence is not evidence of no effect [8, 9]. Current guidelines for standardized care of diabetic patients recommend annual screening for high risk feet. Those identified as high risk should receive enhanced and focused foot care education [10].

Although there is a large amount of literature on diabetic foot and the importance of foot care, there are limited published data on knowledge and practices of foot care among diabetic patients in Sub-Saharan Africa, specifically in Tanzania. Thus, this study was conducted to determine the current prevalence of DFU and to assess the knowledge and practices of foot care among patients who attend public diabetic clinics in Dar es Salaam, Tanzania. The information obtained will inform the current situation in relation to diabetic foot prevention strategies, and will help to improve quality of care for diabetic patients to reduce the burden associated with diabetes foot complications. Educating patients is likely to be effective if we are aware of their current knowledge and practices on foot care.

## Methods

This was a cross-sectional descriptive study, which included 404 patients from public diabetic clinics in Dar es salaam, Tanzania. Data was collected in 2007 from diabetic clinics at MNH and the three municipal hospitals in Dar es Salaam, namely Mwananymala, Amana, and Temeke. A systematic sampling technique was used to select participants in each clinic which was visited once a week. No patient declined to join the study, patients who could not sign consent form because of being under age, were excluded from the study. Patients, who agreed to enter the study but could not wait to have their feet examined, were given an appointment on a later date.

A standard questionnaire was used to enquire about knowledge and practice of foot care. Knowledge was assessed through open-ended questions, without multiple choice answers, as suggested by K. Kaliyaperumal in the guideline for conducting a knowledge, attitude, and practice study [11]. Patients were categorized according to their response on pre-determined answers. Foot self-care practices were adopted from the Summary of Diabetes Self-Care Activities (SDSCA) measure, which identifies the number of days in the past week a person has performed diabetes self-care activities [12]. Each subject underwent foot examination to identify PN, PVD, DFU, or any other pathology that would be used to categorize the feet into risk categories.

Modified Neuropathy Disability Score (NDS) [13] was used to assess PN, whereby absent pain, vibration, and pressure senses were assigned 1 point each and 0 point where they were present. Ankle reflexes were assigned 2, 1, and 0 points for absent, present with reinforcement, and present without reinforcement, respectively. Severity of PN was graded after summation of all the assigned points and classified as no neuropathy (score 0), mild neuropathy (score 1–3), moderate neuropathy (score 4–7) and sever neuropathy (score > 7). Total score of Ankle brachial pressure index (ABPI) was measured using a handheld laser Doppler machine with ABPI of 0.9 as a cutoff point [14]. PVD was defined as ABPI of < 0.9; arterial calcification was defined as ABPI of > 1.3.

Foot risk status was assigned using IDF global guideline for type 2 diabetes, where “no added risk” defines a foot with no any risk factor, “at risk” foot has one risk factor without previous history of DFU or amputation and “high risk” foot has more than one risk factor or previous history of DFU or amputation [15].

### Statistical analysis

All data were analyzed using SPSS statistical package version 14. Comparison between two groups was done by using Pearson correlation (chi-square) and Fisher’s exact test for categorical variables. Univariate and multivariate logistic regressions were done to determine odds ratios. For continuous variables, student’s *t*-test or analysis of variance was used to assess difference between groups. For all these statistics, a p-value of less than 0.05 was considered significant.

### Ethical considerations

Ethical approval was granted by the Research and Publications Ethics committee of the Muhimbili University of Health and Allied Sciences. Permission to collect data was granted from the hospitals authorities. Consent forms were written in “Swahili” language, patients were given the consent to read and sign, and those who could not read it was explained to them and made a thumb print. To maintain confidentiality, all questionnaires were coded and did not bear patients names.

## Results

### Magnitude of risk factors

A total of 404 patients were included in this study, of which 55.4 % were female. Demographic and socioeconomic characteristics of the population are shown in Table 1. Point prevalence of DFU was 15.3 % (95 % CI: 2.17–5.92). Socioeconomic status did not differ between patients with DFU and those without DFU.

**Table 1:** Demographic and socioeconomic characteristics of diabetic patients by presence or absence of diabetic foot ulcer

Characteristics	Total population	DFU present	No DFU	p-value
	N = 404 (%)	N = 62	N = 342	*X* ^2^/Fisher’s test
Age (mean ± S.D)	53.6 ± 12.7	56.4 ± 13.1	53.1 ± 9.9	0.060
Gender				
Female	224 (100)	33 (14.7)	191 (85.3)	0.702
Male	180 (100)	29 (16.1)	151 (83.9)	
Marital status				
Married	305 (100)	49 (16.1)	256 (83.9)	0.455
Never married	28 (100)	2 (7.1)	26 (92.9)	
Previously married	71 (100)	11 (15.5)	60 (84.8)	
Education level				
No formal education	46 (100)	10 (21.7)	36 (78.3)	0.519
Primary education	253 (100)	35 (13.8)	218(86.2)	*0.715
Secondary and vocational	87 (100)	14 (16.1)	73 (83.9)	
Post-secondary education	18 (100)	3 (16.7)	15 (83.3)	
Smoking				
Currently smoking	13 (100)	3 (23.1)	10 (76.9)	0.564
Past smoker	78 (100)	13 (16.7)	65 (83.3)	
Never smoked	313 (100)	46 (14.7)	267 (85.3)	
Alcohol Intake				
Currently drinking	42 (100)	1 (2.4)	41 (97.6)	0.048
Past drinker	142 (100)	24 (16.9)	118 (83.1)	
Never drank alcohol	220 (100)	37 (16.8)	183 (83.2)	

*p-value for trend

Table 2 demonstrates the magnitude of risk factors in univariate and multivariate analysis. In univariate logistic regression analysis, age > 55 years, insulin treatment, and PN all increased the likelihood of having DFU. Alcohol intake was associated with a lower likelihood of DFU. Variables with p-value of < 0.1 were further analyzed in the multivariate logistic regression model. In multivariate analysis, PN and insulin treatment were significant predictors of DFU. Patients with moderate PN were eight times more likely to have DFU, and those with severe neuropathy were 24 times more likely to develop ulcers. Patients on insulin treatment were twice more likely to have DFU than those on oral or non-pharmacological management.

**Table 2:** Univariate and multivariate analysis of predictors of diabetic foot ulcers in diabetic patients (N = 404)

Patient characteristics	Univariate	p-value	Multivariate	p-value
	O.R (95 % CI)		O.R (95 % CI)	
Age:				
< 55	1.00	0.022		0.473
>55	1.91 (1.10–3.30)			
Gender:				
Female	1.00	0.702		
Male	0.90 (0.52–1.55)			
Smoking:				
Never	1.00	0.413		
Current	1.74 (0.46–6.57)	0.664		
Past smoker	1.16 (0.59–2.78)			
Alcohol:				
Current	1.00	0.041		0.322
Past drinker	8.34 (1.09–63.60)	0.040		0.718
Never	8.29 (1.10–62.17)			
Education:				
No formal education	1.00	0.172		
Primary education	0.58 (0.26–1.27)	0.422		
Secondary education	0.69 (0.28–1.71)	0.651		
Post-secondary	0.72 (0.17–2.99)			
Treatment:				
Oral/non-pharmacologic	1.00	0.013	2.25 (1.17–4.32)	0.015
Insulin	2.04 (1.16–3.59)			
PVD:				
No	1.00	0.660		
Yes	1.18 (0.57–2.4)			
PN:				
No neuropathy	1.00	0.387	1.00	0.420
Mild neuropathy	2.03 (0.41–10.1)	<0 .001	1.94 (0.4–9.7)	<0.001
Moderate neuropathy	8.11 (3.3–19.9)	<0.001	8.14 (3.3–20.2)	<0.001
Severe neuropathy	23.46 (10.2–54.0)		24.19 (10.4–56.3)	
Previous DFU:				
No	1.00	<0.001		0.185
Yes	3.23 (1.85–5.6)			
Duration of DM:				
< 5 year	1.00	0.208		0.552
5–10 year	1.49 (0.8–2.7)	0.068		0.962
>10 year	1.94 (0.9–3.9)			
Foot deformity:				
No	1.00	0.052		0.887
Yes	2.04 (0.9–4.2)			

### Knowledge of foot care

Knowledge of diabetic foot risk factors and foot care among the study population is shown in Table 3. The total score for all knowledge questions was 23. Scores from patients’ responses ranged from 0–23, with a mean score of 11.2 ± 6.4 S.D (95 % CI: 10.58–11.83). The mean score was similar among patients with DFU and those without DFU. Higher level of education, longer duration of T2DM, and having received information on foot care were associated with higher mean scores.

**Table 3:** Mean scores of knowledge on diabetes foot care among diabetic patients

Characteristics	Total population N = 404	Mean (SD)	p-value
Age			
<55	211	11.5 (6.4)	0.343
>55	193	10.9 (6.3)	
Gender			
Female	224	11.0 (6.7)	0.435
Male	180	11.5 (6.0)	
Education level			
No formal education	46	8.3 (6.1)	0.002
Primary education	253	11.3 (6.4)	
Secondary	87	12.7 (6.1)	
Post-secondary	18	10.1 (6.8)	
Duration of T2DM (years)			
<5	198	10.2 (6.7)	0.002
5–10	135	12.7 (5.7)	
>10	71	11.2 (6.1)	
Received advice on foot care			
Yes	194	14.1 (5.3)	<0 .001
No	210	8.0 (6.1)	
Source of information (who received advice on foot care)			
Doctor	30	14.8 (4.9)	0 .538
Nurse	152	13.6 (5.4)	
Others	12	13.7 (6.4)	
Previous history DFU			
Yes	107	11.6 (5.9)	0.490
No	297	11.1 (6.6)	
Current DFU			
Yes	64	11.0 (6.0)	0.732
No	340	11.3 (6.5)	

In this study, 194 patients (48.0 %) had received information about foot care. Duration of diabetes had no effect on whether patients received information or not (p = 0.158). The majority of patients received education from nurses (83.5 %); only 16.6 % received foot care information from doctors; and 6.2 % were educated by other sources, such as the media.

### Foot care practices

Table 4 summarizes foot care practices for study patients by risk category. Foot self-inspection was done regularly (6–7 days a week) by 37.9 % of patients, the majority of whom already had DFU.

**Table 4:** Foot care practices of diabetic patients at the time of interview by risk category

Practices	Total	No added risk	At risk	High risk	DFU	p-value
	N = 404	N = 136	N = 108	N = 98	N = 62	
Foot care practices performed for 6–7 days						
Inspecting feet	153 (37.9)	57 (41.9)	40 (37.0)	33 (33.7)	23 (37.1)	0.631
Inspecting shoes	149 (36.9)	58 (42.6)	41 (38.0)	31 (31.6)	19 (30.6)	0.241
Washing feet	378 (92.8)	127 (93.4)	101 (97.2)	85 (89.8)	59 (95.2)	0.573
Drying between toes	213 (52.7)	77 (56.6)	58 (53.7)	48 (49.0)	30 (48.4)	0.596
Not soaking feet	374 (92.6)	129 (94.9)	99 (91.7)	94 (95.9)	53 (85.5)	0.091
Walking barefoot:						
Outside the house	236 (58.4)	84 (61.8)	60 (55.6)	53 (54.1)	39 (62.9)	0.699
Inside the house	53 (13.1)	18 (13.2)	14 (13.0)	17 (17.3)	4 (6.5)	0.493
Use of sharp instruments to cut nails	351 (86.9)	114 (83.8)	91 (84.3)	89 (90.8)	57 (91.9)	0.213

Table 5 shows that patients who had at least one foot examination performed by a doctor were more likely to care for their feet than those who had not. However, only 111 patients (27.5 %) reported having their feet examined by a doctor at least once since their initial diagnosis.

**Table 5:** Foot care practices of patients by whether they had feet examined by doctor or not

Foot care practices done in 6–7 days and foot risk behaviors	Feet examined	Feet not examined	p-value
	N = 111	N = 210	
Inspecting feet	58 (52.3)	95 (32.4)	<0.001
Inspecting shoes	53 (47.7)	96 (32.8)	0.005
Washing feet	107 (96.4)	268 (91.5)	0.087
Drying between toes	71 (64.0)	142 (48.5)	0.005
Not soaking	94 (84.7)	280 (95.5)	<0.001
Never walking barefoot	48 (43.2)	67 (22.9)	<0.001
Use of sharp instruments to cut nails	11 (9.9)	42 (14.3)	0.240

## Discussion

### Risk factors for diabetic foot ulcer

Among persons with diabetes, the lifetime risk of developing DFU has been estimated to be 15 %; however, recent studies have shown that lifetime incidence could be as high as 25 % [1, 5]. In this study, the prevalence of DFU among patients attending public diabetic clinics in Dar es Salaam was 15 %. A similar prevalence (15 %) was reported by Abbas et al. among diabetic patients admitted to MNH from 1997 to 1998 [16], which raises concerns that more efforts are needed to prevent DFU.

PN has been described as the most common component cause in the pathway to diabetic foot ulceration [17]. For the past 10 years, published data on prevalence of PN in Africa has ranged from 25 % in Tanzania to 84 % in Algeria [2]. Part of reason for the massive difference in the prevalence of peripheral neuropathy in Africa is the heterogeneity of the studies and differences in diagnostic criteria. In this study, the prevalence of PN was 44 %, while the prevalence of PN among patients with foot ulcer was 94 %. Patients with moderate PN were eight times more likely to have foot ulcers, while those with severe PN were 24 times more likely than those with no neuropathy. These findings signify the importance of education on caring for insensate feet to prevent minor trauma, which is a significant factor in the development of foot ulcers. Patients need to be educated on the importance of glycaemic control in order to prevent long term diabetes complications which include PN. Emphasis should be placed on older patients and those with longer duration of diabetes since they are more prone to have PN and are likely to have other complications, such as retinopathy, that may put them at even greater risk of injury [18, 19].

PVD was present in 15 % of patients, and was not associated with DFU in this study. Contrary to this finding, Boyko et al. identified ABPI to be an independent risk factor for developing foot ulcer in the Seattle diabetic foot study [20].

### Knowledge of foot care

The importance of foot care knowledge in preventing foot ulcers in diabetic patients is a widely accepted fact; however, over half of the diabetic patients in this study reported they had never received information regarding foot care. Level of knowledge on foot care was similar among patients with DFU and those without DFU. Lack of knowledge about diabetic foot among health care providers, shortage of staff, and overcrowding of clinics can partly explain these findings. Most clinics are run once or twice per week—with daily attendance of about 50 patients—with two nurses and two doctors. To arrange an individualized educational session is usually difficult, and providing an appointment on a separate day incurs a financial burden on the patient. Education on foot care is usually given universally without being individualized according to the foot risk of the patients.

The mean knowledge score was influenced by level of education, duration of diabetes, and advice on foot care. Patients with no formal education had lower mean scores, as did those with diabetes duration of less than 5 years and those who had not received advice on foot care. Patients with longer duration of diabetes are more likely to have repeated education sessions, which may favor their knowledge scores. Khamseh et al. also found that low knowledge scores were attributed to low level of education and to not receiving advice on foot care; however, the duration of diabetes had no effect in his study [21]. People with a higher education level are more likely to read and to obtain information regarding foot care in addition to any information provided to them in the clinics.

In the diabetic outpatient clinics, regular educational classes are provided on a day separate from the clinic day. These classes are open to all patients with diabetes, but emphasis is placed on attendance by newly diagnosed patients. Patients may only attend once, shortly after they are diagnosed, and thereafter not see the need to attend. Moreover, patients may feel the education is not worth the cost (i.e., patients have to incur the cost of transportation to/from the clinic on a separate day). Those with foot problems, especially painful PN, may not be able to visit the clinic frequently. These patients may opt to make just one trip, often the one in which they will be getting prescriptions for their medications. For these reasons, Ward et al. recommended a flexible schedule for diabetes education, offering education at any time for the maximum convenience of patients rather than focusing on health care provider’s convenience, preferably integrating it into normal consultation [22]. Educational programs should also address psychological and cultural factors that often underlie self-care behavior [23].

### Foot self-care practices

In this study, foot self-care practices were not performed by many patients, even in the groups at high risk for developing foot ulcers. The majority of patients did not inspect their feet regularly or inspect the insides of their shoes. Risky behaviors, such as cutting toenails with sharp instruments (e.g., razor blade or knife) were performed by over 80 % of study patients. These behaviors can cause minor injuries especially if a patient has PN or retinopathy as part of diabetes complications.

Foot self-care was practiced similarly among all risk categories of patients, but improved among those who had received advice on foot care and those whose feet have been examined by a doctor at least once. Bell et al. made similar observations in evaluating long-term diabetes self-management among an elderly population in the United States [24]. They found the following patients were more likely to practice foot self-care than those who did not meet these criteria: patients who had received advice on foot care, and patients who had their feet examined by a doctor or were shown by a doctor how to examine their feet. This fact should alert physicians treating patients with diabetes to always have interest in evaluating the patients’ feet as the physician’s attention and behavior has a positive effect on their patients’ foot care practice.

## Conclusions

The prevalence of diabetic foot ulcers is high among patients attending public diabetic clinics in Dar es Salaam. Peripheral neuropathy is the major risk factor for foot ulcer in this population. Avoidance of injuries to an insensate limb and appropriate foot care practice by both patients and health care providers may reduce DFU. Knowledge of foot care is low among patients with diabetes. Their knowledge can be improved by education and proper foot care modeling by health care providers. It is essential to assess patients’ beliefs and behavior so as to offer education and utilize educational methods that facilitates them to care for their feet efficiently.

There is an urgent need to educate and raise awareness among doctors regarding the importance of identifying risk factors for foot ulcers in patients with diabetes. Knowledge alone however, does not always lead to change in behavior and in Africa there are several other challenges that lead to poor foot care including non-existent podiatry services, lack of programs for health care professionals and lack of surveillance mechanism.

Health care facilities should incorporate foot care services among other routine services being provided to diabetic patients in order to identify patients with risk factors and those who already have foot ulcers. There is a need for continuous education on foot care to improve patients’ knowledge of risks and foot self-care practices.

## Abbreviations

ABPI: Ankle brachia pressure index
IDF: International Diabetes Federation
DFU: Diabetic foot ulcer
MNH: Muhimbili National Hospital
PN: Peripheral neuropathy
PVD: Peripheral vascular diseases
T2DM: Type 2 Diabetes
